# Severe Postpartum Hemorrhage After Vaginal Delivery in an Osteogenesis Imperfecta Type I Patient: A Case Report

**DOI:** 10.1002/ccr3.73107

**Published:** 2026-07-03

**Authors:** Madeline Dow, Aariya Srinivasan, Sabry Ayad

**Affiliations:** ^1^ Department of Anesthesiology Research Cleveland Clinic Foundation Cleveland Ohio USA; ^2^ Department of Anesthesiology Outcomes Research Consortium Cleveland Ohio USA; ^3^ Cleveland Clinic, Fairview Hospital Cleveland Ohio USA

**Keywords:** anesthesia, genetics and genomics, obstetrics/gynecology, perinatal medicine

## Abstract

Pregnant patients with osteogenesis imperfecta type I are at increased risk of obstetric and hemorrhagic complications. Early multidisciplinary planning and anesthesiology involvement are essential. Severe postpartum hemorrhage may result from uterine atony and tissue fragility, requiring prompt uterotonic therapy, surgical intervention, blood products transfusion, and individualized analgesic management to optimize maternal outcomes.

## 1 Introduction

Osteogenesis imperfecta (OI) is a heterogeneous, inherited connective tissue disorder associated with bone fragility and recurrent fractures. In addition to skeletal fragility, individuals with OI may exhibit bone deformity, reduced bone mass, growth retardation, dental abnormalities, blue sclerae, hearing loss, ligament laxity, reduced respiratory function, and cardiac valvular regurgitation [1, 2]. OI is most commonly caused by autosomal dominant mutations in the *COL1A1* or *COL1A2* genes, which encode the alpha‐1 and alpha‐2 chains of type I collagen. Type I collagen is a major structural component of bone and connective tissue extracellular matrix. OI type I, the most common clinical subtype, accounts for approximately 85%–90% of affected individuals [1, 3]. The type I subtype is generally characterized by less severe bone fragility, with variable fracture rates, minimal skeletal deformity, and normal or near‐normal stature [1, 3].

The estimated prevalence of OI is approximately 1 in 15,000–20,000 individuals [2, 4]. Within this population, pregnancy represents a period of heightened physiologic demand characterized by increases in ligament laxity, bone turnover, and cardiopulmonary function [4]. Compared with pregnant individuals without OI, patients with OI have been reported to have higher rates of obstetric complications, including antepartum hemorrhage, malpresentation, preterm delivery, cesarean delivery, congenital abnormalities, and premature rupture of membranes [4, 5]. Despite the increased risk of complications, current studies do not demonstrate a consistent association between OI and hypertensive conditions, such as preeclampsia or eclampsia [4, 5]. Given the limited literature on OI in pregnancy and the complexity of peripartum physiologic considerations, early anesthesiology evaluation is recommended to anticipate peripartum challenges [3]. We present a case of severe postpartum hemorrhage following vaginal delivery in a patient with OI type I, with emphasis on the contribution of both uterine atony and tissue fragility to severe postpartum hemorrhage, as well as the importance of early multidisciplinary management in facilitating timely recognition and treatment.

## 2 Case History and Examination

A 30‐year‐old primigravida with osteogenesis imperfecta (OI) type I at 33 weeks and 3 days gestation presented with elevated blood pressure. On presentation, vital signs were notable for sustained hypertension, with otherwise stable heart rate and oxygen saturation. Her medical history was significant for chronic hypertension, preeclampsia with severe features, gestational diabetes mellitus, class III obesity, and anxiety disorder. Home medications included labetalol 100 mg twice daily, fluoxetine 40 mg daily, neutral protamine Hagedorn insulin 8 units nightly, and insulin aspart 4 units with meals. She reported a history of six fractures, with the most recent nonoperative fracture involving the L1 vertebra occurring 7 years prior. The patient noted chronic lower back pain without associated neurologic symptoms or mobility limitation. She denied headache, visual changes, shortness of breath, chest pain, vaginal bleeding, or uterine contractions. Physical examination findings included blue sclerae and intact dentition without evidence of dentinogenesis imperfecta. She had no apparent limb deformities or mobility limitations. Airway examination was notable for Mallampati class III anatomy. Her height was 172.7 cm and weight was 159.7 kg, corresponding to a body mass index of 53.52 kg/m^2^.

## 3 Investigations and Diagnosis

Multidisciplinary consultation with obstetrics and anesthesiology was obtained, and the patient was diagnosed with preeclampsia with severe features based on persistent hypertension, prompting a recommendation for induction of labor. Laboratory evaluation demonstrated platelet count of 310 × 10^9^/L, hemoglobin 12.8 g/dL, normal liver transaminases (AST 19 U/L, ALT 20 U/L), and creatinine 0.51 mg/dL. Given the patient's history of OI type I, operative vaginal delivery using forceps or vacuum assistance was avoided because of concern for maternal injury, and spontaneous vaginal delivery was pursued. Review of spinal imaging revealed no anatomic contraindications to neuraxial anesthesia, and both epidural and spinal techniques were considered appropriate.

## 4 Management

Labor was induced with intravenous oxytocin and artificial rupture of membranes. Six hours after induction, a lumbar epidural catheter was placed at the L3–L4 interspace in the sitting position using a midline approach for labor analgesia. At the time of epidural placement, the patient was hemodynamically stable, with blood pressure of 143/81 mmHg, heart rate of 80 beats per minute, respiratory rate of 16 breaths per minute, and oxygen saturation of 98% on room air. Epidural catheter placement was confirmed with a 3 mL test dose of 1.5% lidocaine with epinephrine (1:200,000) to exclude inadvertent intravascular placement. Analgesia was then established with epidural 0.125% bupivacaine with fentanyl 5 mcg/mL in divided boluses totaling 14 mL and maintained with a continuous epidural infusion of 0.1% bupivacaine with fentanyl 2 mcg/mL at 8 mL/h, later increased to 12 mL/h, with 5 mL patient‐controlled boluses available every 15 min as needed.

The patient progressed well to complete cervical dilation and a liveborn female infant was delivered with a birth weight of 3106 g and Apgar scores of 7 at 1 min and 8 at 5 min. The placenta was delivered spontaneously and intact shortly after delivery. Immediately following placental delivery, approximately 900 mL of blood loss was noted. The uterine fundus was atonic. Bimanual uterine massage was initiated and the patient received a standard oxytocin bolus, carboprost, 1000 μg of rectal misoprostol, and 1 g of tranexamic acid.

Persistent bleeding prompted further evaluation, which revealed a large vaginal laceration, a left sulcal laceration, a right labial laceration, and a right mediolateral second‐degree perineal laceration with intact skin. During attempted repair, the patient reported severe perineal pain rated 8/10 on the Visual Analog Scale, which prompted redosing of epidural analgesia. Despite uterotonic therapy, uterine atony persisted, and repeated bedside laceration repairs were complicated by reaccumulating hematomas and recurrent skin tears. A Jada device was placed for hemorrhage management. As bleeding continued, estimated blood loss increased to approximately 1800 mL. A second intravenous line was placed, and packed red blood cells were requested. Despite these interventions, hemorrhage persisted and the patient was transferred to the operating room for improved visualization and repair. Vaginal packing was placed for hemostasis during transfer. In the operating room, a right mediolateral episiotomy was performed to visualize the accumulating internal hematomas. The area was sutured. Intraoperatively, the patient received two units of packed red blood cells. Total quantified blood loss from delivery and operative intervention, determined by direct measurement of collected blood and gravimetric assessment of blood‐soaked materials, was approximately 2410 mL.

## 5 Outcome and Follow‐Up

The patient reported severe perineal pain (9/10 on the Visual Analog Scale) immediately postpartum despite receiving 5 mg oral oxycodone. One dose of 0.2 mg intravenous hydromorphone was administered with minimal relief, so the epidural infusion was restarted and maintained for ongoing pain control. Postdelivery blood pressures were labile, averaging around 109/55 mmHg. Hemoglobin dropped from 10.4 to 9.5 g/dL with all other laboratory values remaining within normal limits, and the patient received two additional units of packed red blood cells. Following removal of the Jada device and vaginal packing on postpartum day 1, the patient underwent further imaging due to concern for pelvic hematoma. Computed tomography angiography of the pelvis demonstrated a hematoma measuring 9 × 15 cm without evidence of active contrast extravasation. Consultation with interventional radiology and gynecologic oncology determined that no procedural intervention was indicated.

The patient received a brief course of magnesium sulfate on postpartum day 1, which was discontinued prematurely due to hypotension. By postpartum day 3, the patient reported adequate pain control with acetaminophen 1000 mg every 6 h as needed. Blood pressure remained stable on labetalol 200 mg every 8 h. Follow‐up laboratory studies demonstrated hemoglobin of 7.9 g/dL, platelet count of 222 × 10^9^/L, PT/INR 0.9, aPTT 24 s, and fibrinogen 553 mg/dL. Postpartum glucose was normal, and insulin was discontinued. A 2‐h oral glucose tolerance test was scheduled for the postpartum follow‐up visit. The patient was subsequently discharged in stable condition.

## 6 Discussion

Osteogenesis imperfecta (OI) is a heterogeneous group of inherited connective tissue disorders caused by mutations in collagen‐related genes, most commonly involving defects in the synthesis or structure of type I collagen and characterized by skeletal fragility and multisystem involvement [1, 2, 3]. In OI type I, defects in *COL1A1* or *COL1A2* lead to reduced or abnormal collagen production and are associated with a relatively mild phenotype compared with other OI subtypes, although fracture frequency and clinical severity vary widely among affected individuals [1, 3]. Current literature guiding obstetric and anesthetic management of patients with OI remains limited, particularly in pregnancy. Available evidence is largely derived from case reports, narrative reviews, and population‐based studies [3, 4, 6]. A review of OI in pregnancy noted an absence of evidence regarding specific medical therapies for the management of OI during pregnancy, underscoring the limited guidance available for clinical management in this population [3].

Pregnancy in individuals with OI is associated with an increased risk of adverse outcomes compared with the general obstetric population. Complications in patients with OI include antepartum hemorrhage, malpresentation, preterm delivery, cesarean delivery, congenital abnormalities, and premature rupture of membranes [4, 5]. In contrast, fracture risk in pregnant individuals with OI does not appear to be significantly increased compared with the nonpregnant state [4]. In addition to these considerations, bleeding risk is also an important concern in patients with OI. Clinical features described in OI include platelet dysfunction and generalized tissue fragility involving bone, joints, blood vessels, and skin, which may contribute to hemorrhagic risk [3]. Type I collagen plays a key role in the structure of fetal membranes and in tissue repair through promotion of M2 macrophages. In OI, this pathway may be defective, allowing microlesions to persist [4]. Accordingly, a narrative review of neuraxial and regional anesthesia in surgical patients with OI reported an increased risk of bleeding and hemorrhagic complications due to vascular fragility and impaired hemostasis from defective type I collagen [6]. In addition, the patient's type I collagen defect may have contributed to uterine atony through impaired uterine extracellular matrix support, as collagen provides tensile strength and structural integrity to uterine tissue and is present within the endometrium and myometrial smooth muscle [7]. During pregnancy, uterine collagen cross‐linking increases to support normal myometrial function, and abnormalities in type I collagen may therefore impair coordinated postpartum uterine contraction. In the present case, severe postpartum hemorrhage resulted from uterine atony and extensive vaginal and perineal lacerations with hematoma formation, which required uterotonic therapy, tranexamic acid administration, operative repair, and blood transfusion.

Anesthetic management in patients with OI requires careful preoperative assessment and planning. Abnormalities in airway anatomy have been described, with associated difficulty during intubation and risk of spinal fractures during airway manipulation [1, 3]. Structural spinal abnormalities, such as scoliosis and altered vertebral anatomy, may further complicate anesthetic management [3, 6]. Additionally, given skeletal fragility, careful patient handling, positioning, and transfers are recommended to minimize fracture risk [1].

Neuraxial and regional anesthesia may offer advantages in obstetric patients with OI but can present technical challenges related to short stature, scoliosis, and vertebral deformities [6]. As such, anesthesiology evaluation during early pregnancy is recommended to anticipate potential airway, spinal, and positioning challenges [3]. Continuous spinal anesthesia with an epidural catheter has been described as a safer alternative compared with general anesthesia or single‐shot spinal anesthesia in select patients with OI [3]. In our patient, predelivery anesthesiology consultation and multidisciplinary review of spinal imaging identified no anatomic contraindications to neuraxial anesthesia, and labor analgesia was successfully managed with a lumbar epidural catheter without anesthetic complications.

One review cautions against routine induction of labor in patients with OI due to difficulty predicting the effects of induced uterine contractions on structurally abnormal collagen [3]. To reduce delivery‐related trauma, cesarean delivery, is suggested to reduce the risk of pelvic or vertebral fractures and uterine rupture [3]. In this case, induction of labor was pursued despite these considerations due to preeclampsia with severe features following multidisciplinary evaluation of maternal and fetal risks and benefits. After consultation with maternal‐fetal medicine and anesthesiology, vaginal delivery was considered an appropriate option given the patient's relatively mild OI phenotype, absence of prior pelvic fractures, and lack of evidence of fetal fractures. Although cesarean delivery may reduce certain obstetric risks in patients with OI, it would also have been associated with potential disadvantages in this patient, including prolonged postoperative recovery and an increased risk of wound complications in the setting of class III obesity and gestational diabetes mellitus. In this case, multidisciplinary planning facilitated an individualized delivery approach that balanced the risks associated with both vaginal and cesarean delivery.

Pain management is another important consideration in patients with OI. A multicenter study reported chronic pain in approximately 40% of individuals with OI compared with 20% of the general adult population, with nonsteroidal anti‐inflammatory drugs as the most common treatment, followed by bisphosphonates and physical therapy [8]. In patients with OI type I, female sex and higher body weight were identified as predictors of chronic pain [8]. Repeated fractures and prior exposure to opioids, benzodiazepines, and muscle relaxants may also contribute to tolerance and complicate perioperative analgesia [6]. Postpartum pain in our patient was adequately managed with nonopioid analgesics after delivery.

This case adds to the limited literature on OI in pregnancy by describing severe postpartum hemorrhage in which uterine atony occurred alongside extensive vaginal and perineal tissue injury. While the patient's type I collagen defect may have contributed to impaired postpartum uterine contraction, the clinical course was also marked by recurrent tissue tearing and hematoma formation during attempted repair. These features complicated hemorrhage control despite standard uterotonic therapy and required escalation to operative repair, transfusion, and continued postpartum evaluation. In this context, early anesthesiology involvement and multidisciplinary communication were important in anticipating peripartum risks, supporting analgesic management, and coordinating escalation of care.

As a single case report, this report cannot establish a definitive relationship between OI type I and uterine atony. Moreover, the patient's postpartum hemorrhage was likely influenced by multiple contributing factors, including tissue fragility and coexisting obstetric comorbidities. These limitations should be considered when interpreting the clinical associations described in this report.

## 7 Conclusion

This case highlights the complexity of peripartum management in pregnant patients with osteogenesis imperfecta type I. Individuals with OI have an increased risk of obstetric complications, including antepartum hemorrhage, preterm delivery, and postpartum complications. Current evidence remains limited, with no standardized guidelines for management. Multidisciplinary care with early anesthesiology involvement is essential to address airway challenges, spinal deformities, fracture prevention, bleeding risk, and pain management in pregnant patients with OI. The limited literature on OI in pregnancy underscores the need for further research to establish evidence‐based and standardized management guidelines for this population.

## Author Contributions

**Madeline Dow:** conceptualization, visualization, writing – original draft, writing – review and editing. **Aariya Srinivasan:** conceptualization, visualization, writing – original draft, writing – review and editing. **Sabry Ayad:** conceptualization, supervision, writing – review and editing.

## Funding

The authors have nothing to report.

## Consent

Written informed consent was obtained from the patient whose case details are written in the study to publish this report in accordance with the journal's patient consent policy.

## Conflicts of Interest

The authors declare no conflicts of interest.

## Data Availability

The data supporting this report's findings are available on request from the corresponding author. The data are not publicly available due to privacy or ethical restrictions.
